# IMMUNOSARC II Master Trial: Phase II Study of Sunitinib and Nivolumab in Clear Cell Sarcoma Cohort

**DOI:** 10.34133/cancomm.0015

**Published:** 2026-03-12

**Authors:** Javier Martin-Broto, Sandra J. Strauss, Emanuela Palmerini, Claudia Valverde, Ana Sebio, Andres Redondo, Silvia Stacchiotti, Giovanni Grignani, Sandra Aliberti, Roberto Diaz-Beveridge, Enrique Gonzalez Billalabeitia, Josefina Cruz, Irene Carrasco-Garcia, Toni Ibrahim, Juan Diaz-Martin, Carmen Salguero-Aranda, Antonio Gutierrez, Empar Mayordomo-Aranda, Rafael Ramos, Jose Merino, Paola Collini, Roberto Tirabosco, Silvia Bague, Cleofe Romagosa, Maria Augusta Carrera, Patricio Ledesma, Nadia Hindi, David Silva Moura

**Affiliations:** ^1^ Department of Medical Oncology, Fundación Jiménez Díaz University Hospital, Madrid, Spain.; ^2^ University Hospital General de Villalba, Madrid, Spain.; ^3^ Fundación Jiménez Díaz Health Research Institute, Autonomous University of Madrid, Madrid, Spain.; ^4^ Department of Medical Oncology, University College London Hospital, London, UK.; ^5^ Department of Medical Oncology, Istituto di Ricovero e Cura a Carattere Scientifico, Istituto Ortopedico Rizzoli, Bologna, Italy.; ^6^ Department of Medical Oncology, Sylvester Comprehensive Cancer Center, Miller School of Medicine, University of Miami, Miami, FL, USA.; ^7^ Department of Medical Oncology, University Hospital Vall d’Hebron, Barcelona, Spain.; ^8^ Department of Medical Oncology, Sant Pau Hospital, Barcelona, Spain.; ^9^ Department of Medical Oncology, University Hospital La Paz, Madrid, Spain.; ^10^ Medical Oncology Department, Fondazione Istituto di Ricovero e Cura a Carattere Scientifico, Istituto Nazionale dei Tumori, Milan, Italy.; ^11^ Medical Oncology Department, Candiolo Cancer Institute, Fondazione Piemontese per l’Oncologia-Istituto di Ricovero e Cura a Carattere Scientifico (FPO-IRCCS), Candiolo, Italy.; ^12^ Department of Medical Oncology, University Hospital La Fe, Valencia, Spain.; ^13^ Department of Medical Oncology, University Hospital 12 de Octubre, Madrid, Spain.; ^14^ Department of Medical Oncology, Canarias University Hospital, La Laguna, Spain.; ^15^ Department of Medical Oncology, University Hospital Virgen del Rocio, Seville, Spain.; ^16^ Institute of Biomedicine of Seville, Spanish National Research Council-University of Seville, Department of Pathology, University Hospital Virgen del Rocio, Seville, Spain.; ^17^ Biomedical Research Networking Center in Cancer (CIBERONC), Carlos III Health Institute, Madrid, Spain.; ^18^ Department of Hematology, University Hospital Son Espases, Palma de Mallorca, Spain.; ^19^ Department of Pathology, University Hospital La Fe, Valencia, Spain.; ^20^ Department of Pathology, University Hospital Son Espases, Palma de Mallorca, Spain.; ^21^ Department of Pathology, Fundación Jiménez Díaz University Hospital, Madrid, Spain.; ^22^ Advanced Diagnostic Department, Fondazione Istituto di Ricovero e Cura a Carattere Scientifico Istituto Nazionale dei Tumori, Milan, Italy.; ^23^ Department of Pathology, University College London Hospital, London, UK.; ^24^ Department of Pathology, Sant Pau Hospital, Barcelona, Spain.; ^25^ Department of Pathology, University Hospital Vall d’Hebron, Barcelona, Spain.; ^26^ Sofpromed Investigación Clínica, Palma de Mallorca, Spain.

## Abstract

**Background:** Clear cell sarcoma (CCS) is an ultrarare sarcoma driven by a specific chromosomal translocation, most commonly the EWS RNA binding protein 1–activating transcription factor 1 fusion (*EWSR1::ATF1*), for which chemotherapy shows limited activity, with a median progression-free survival (PFS) of approximately 3 months in retrospective series. In the present trial, a CCS cohort was selected based on signals of activity observed in the IMMUNOSARC I phase I/II trial evaluating nivolumab in combination with sunitinib in sarcomas. **Methods:** Patients aged 12 to 80 years with advanced, progressive, and measurable CCSs were enrolled after central pathology review, and molecular confirmation of an *EWSR1* rearrangement was required. Sunitinib was administered at 37.5 mg/d during the first 2 weeks and then at 25 mg/d along with nivolumab at 240 mg every 2 weeks. The primary end point was the 6-month PFS rate, defined under the null (H_0_) and alternative (H_1_) hypotheses as 25% and 55%, respectively. Under Simon’s 2-stage minimax design (α = 0.05, power = 0.90), a minimum of 10 of 23 patients needed to be progression-free at 6 months. **Results:** At the time of cutoff, 23 patients were evaluable for the primary end point. With a median follow-up of 23.0 months (95% confidence interval [CI], 10.0 to 35.0 months), the 6-month PFS rate was 50.1% (95% CI, 29.1% to 71.1%), while the median PFS was 6.2 months (95% CI, 3.0 to 9.3 months). Of 21 patients who underwent at least 1 radiological assessment, 3 (14.3%) achieved partial response, 14 (66.7%) had stable disease, and 4 (19.0%) had progressive disease. The median overall survival was 17.0 months (95% CI, 95% CI, 5.6 to 28.5 months). The main all-grade drug-related toxicities were lymphocytopenia (46.2%), leukopenia (38.5%), anemia (38.5%), and neutropenia (38.5%). Two grade 4 toxicities were reported: Alanine aminotransferase increased and ischemia (each 3.8%), while 31 grade 3 toxicities occurred, with anemia and lymphocytopenia being the most common (each 23.1%). A higher programmed death-ligand 1 composite score was associated with better PFS: 21.2 months (95% CI, 6.0 to 36.4 months) versus 4.2 months (95% CI, 2.7 to 5.6 months), *P* = 0.045. **Conclusions:** While further studies are needed, initial findings suggest that nivolumab plus sunitinib could be a valuable addition to the current armamentarium for CCS management. **Trial registration:** ClinicalTrials.gov ID NCT03277924 (date of registration: 2017 September 6).

## Background

Clear cell sarcoma (CCS) is an ultrarare sarcoma, with an incidence after population-adjusted rates that range from 0.012 to 0.027 per 100,000 inhabitants between 2000 and 2020 in the United States [1]. Although it is localized at diagnosis in up to 80% of cases, the likelihood of metastasis within the first 5 years after local treatment is high, reaching up to 68% [2]. Typically, CCS affects young individuals around 25 years old, originates from aponeurotic structures and tendons in the lower extremities, and tends to spread to lymph nodes and lungs. This tumor exhibits low sensitivity to classic chemotherapeutic agents and tyrosine kinase inhibitors (TKIs), showing a median progression-free survival (PFS) of 2 months. In one of the largest retrospective series to date, the objective response rate (ORR) of systemic treatments for CCS was generally 0.0%, with limited exceptions observed for doxorubicin at 11.8% (4/34), gemcitabine at 15.4% (2/13), and sunitinib at 30.0% (3/10) [3].

Molecularly, CCS is driven by a reciprocal translocation, with EWS RNA binding protein 1–activating transcription factor 1 fusion (*EWSR1::ATF1*), t(12;22)(q13;q12) in 85% of cases and also the fusion *EWSR1::CREB1*, t(2;22)(q34;q12) in 15% of cases. Both activating transcription factor 1 (*ATF1*) and cyclic adenosine monophosphate response element binding protein 1 (*CREB1*) up-regulate microphthalmia-associated transcription factor (*MITF*), particularly its melanocytic isoform (*M-MITF*), inducing the transcription of several target genes such as c-mesenchymal–epithelial transition factor (*c-MET*), a key driver of proliferation, survival, and metastasis [4]. These connections have been the basis of the first 2 trials conducted in CCS with MET inhibitors. A trial exploring tivantinib reported an ORR of 9.1%, a median PFS of 1.9 months, and a median overall survival (OS) of 5.2 months in 11 enrolled CCS patients [5], and another trial testing crizotinib obtained an ORR of 3.8%, a median PFS of 4.4 months, and a median OS of 9.2 months [6].

Thus, data on the inherent aggressive biology of CCS, along with the limited activity of the tested systemic agents, reflect a clear unmet need in the sarcoma field. Based on signals of activity detected in the IMMUNOSARC I, with 2 partial responses (PRs) of 11 patients diagnosed with CCS, we decided to incorporate a specific CCS cohort in IMMUNOSARC II, exploring the combination of sunitinib and nivolumab in a phase II setting [7]. Tumor angiogenesis not only sustains sarcoma growth but also creates a profound immune barrier, as angiogenic mediators such as vascular endothelial growth factor A actively suppress antitumor immunity [8]. Thus, combining the antiangiogenic agent sunitinib with the immune checkpoint inhibitor nivolumab may be synergistic by simultaneously dismantling vascular-driven immunosuppression and restoring effective immune responses [9].

## Methods

### Study design and participants

This trial enrolled patients aged 12 to 80 with advanced, measurable, centrally confirmed CCS. Other inclusion criteria were Eastern Cooperative Oncology Group (ECOG) performance status of 0 to 1, adequate bone marrow function and hepatic function (aminotransferase ≤2.5 times the upper limit of normal [ULN], total bilirubin ≤1.5 times the ULN, and alkaline phosphatase ≤2.5 times the ULN), creatinine phosphokinase ≤2.5 times the ULN, adequate renal function (serum creatinine of ≤1.5 mg/dl), prothrombin time and INR of <1.5 in the absence of anticoagulant therapy, and left ventricular ejection fraction of ≥50%. Females of childbearing potential had to have a negative serum or urine pregnancy test within 7 d before enrollment and to agree to use birth control measures during study treatment and for 6 months after its completion. Previous antiangiogenic treatment was allowed. Some exclusion criteria were 4 or more previous lines of chemotherapy; previous administration of anti-programmed death-1 (PD-1) or anti-PD-ligand 1 (PD-L1), anti-PD-L2, or anti-cytotoxic T-lymphocyte antigen 4 antibody; previous immune-related adverse event (i.e., after cancer vaccine); active, known, or suspected autoimmune disease; a condition requiring systemic treatment with either corticosteroids (>10 mg of daily prednisone or equivalent) or other immune suppressive medications within 14 d of study administration; pregnancy; or breastfeeding. The full list of inclusion and exclusion criteria is presented in the Supplementary Materials. The patient or legal tutors had to provide written informed consent before performing study-specific procedures. Study procedures were in accordance with guidelines established by the ethics committee of each hospital and with the Declaration of Helsinki. Approval was obtained from the ethics committee of each participating center. Patients were enrolled at 11 hospitals with expertise in sarcoma management in Italy, the United Kingdom, and Spain (Table S1).

### Procedures

Patients received sunitinib 37.5 mg/d during the first 15 d. From then on, sunitinib was administered at 25 mg/d, and nivolumab was administered at a 240-mg flat dose intravenously over 30 min, starting from day 15, every 2 weeks. The protocol (Supplementary Materials) specified a dose of nivolumab of 3 mg/kg for pediatric patients weighing <40 kg. Treatment was maintained up to progression or intolerance.

Dose adjustment criteria are thoroughly detailed in the Supplementary Materials and were applied to any of the drugs to which adverse events were related. Thus, if an adverse event was related only to 1 drug, the other could be maintained. In the case of sunitinib, if a recurrent grade 2 toxicity occurred, then the schedule of 2 weeks on and 1 week off could be adapted to maintain the best dose intensity. The same procedure was applied to hematological toxicity; if grade 3 or intolerable grade 2 toxicity occurred, then the protocol recommended withholding the dose until toxicity was grade ≤2, then resuming the treatment at the same dose level. If the toxicity recurred with grade 3 toxicity or intolerable grade 2, then the schedule 2 weeks on and 1 week off was recommended. Likewise, if grade 4 hematological toxicity appeared, then the protocol recommended withholding the drug until grade ≤2 and then reducing the dose by applying 2 weeks on and 1 week off. In the case of nivolumab, its administration had to be delayed for grade 3 skin toxicity or any grade ≥2 nonskin, drug-related adverse event except grade 2 drug-related fatigue or laboratory abnormalities. Nivolumab had to be delayed for any grade 3 drug-related laboratory abnormality, with some exceptions detailed in the protocol. Guidelines for withdrawing or withholding were also defined for each drug and different toxicities, as detailed in the Supplementary Materials.

### Outcomes

The main end point was the PFS rate at 6 months according to Response Evaluation Criteria in Solid Tumors, version 1.1 (RECIST 1.1). The 6-month PFS rate was defined as the percentage of patients who did not experience progression or death due to any cause in the first 6 months since treatment initiation. Secondary objectives included OS, measured from treatment initiation to the date of the last follow-up. ORR was determined according to RECIST 1.1 and following the central radiological assessment. In addition, the safety profile of this combination was graded and tabulated through assessment of adverse events by using Common Terminology Criteria for Adverse Events version 5.0, and tumor biomarker analysis was planned for correlative studies. For this purpose, a biopsy of tumor tissue was compulsory at baseline, and blood samples were collected at baseline and coincident with every radiological assessment.

### Sample size

The sample size was obtained for the primary end point of the 6-month PFS rate. A 6-month PFS rate of 25% (in line with the previous results of MET inhibitors) [5,6] was considered not promising, whereas a 6-month PFS rate of 55% was considered promising in this population. With a type I error α of 0.05 and a power of 0.90, 23 patients were estimated in this cohort. With Simon’s 2-stage minimax design [10], at least 4 cases over the first 13 patients (stage 1) had to have a PFS rate of 6 months or longer. Then, 10 additional patients would be accrued, up to 23 evaluable patients. If at least 10 patients had a 6-month PFS rate, then further investigation of the drug was considered warranted.

### Targeted RNA sequencing

For patients without complete information on the fusion gene partners from local sites, targeted RNA sequencing was performed. Total nucleic acid was extracted from formalin-fixed paraffin-embedded tissue samples using an Agencourt Formapure Kit (A33341; Beckman Coulter, USA) following the manufacturer’s instructions. The isolated RNA was quantified using a Qubit RNA HS assay kit (Q32852; Thermo Fisher Scientific, USA) in combination with a Qubit 4 fluorimeter (Thermo Fisher Scientific, USA). A total of 200 ng of RNA was used for targeted library preparation using the Archer FusionPlex Sarcoma v2 panel (Integrated DNA Technologies, USA) based on a targeted enrichment method called anchored multiplex polymerase chain reaction. Briefly, RNA was reverse-transcribed using random primers, first-strand complementary DNA (cDNA) was synthesized, and RNA quality was assessed using the Archer PreSeq RNA QC assay. After second-strand cDNA synthesis, end repair, A-tailing, and adapter ligation, cDNA was amplified using 2 rounds of nested polymerase chain reaction using gene-specific primers. Final libraries were quantified with a KAPA Library Quantification Kit (KK4824; Kapa Biosystems, USA) and pooled to equimolar concentration. Libraries were sequenced on an Illumina MiSeq with MiSeq 300v2 reagents (MS-102-2002; Illumina, USA) for paired-end reads, 150-base pair reads, and dual-index reads. Demultiplexed FASTQ files were analyzed using Archer analysis pipeline v7.1.0-14. A minimum of 5 reads with 3 or more unique start sites spanning the break points was set as cutoffs to call fusions.

### Immunohistochemistry

Paraffin-embedded tissue sections (4 μm thick) were baked at 65 °C for 30 min. Antigen retrieval was performed using a PT Link instrument (Agilent, USA) with EDTA buffer (97 °C, 20 min). Endogenous peroxidase activity was quenched by immersing the sections in an aqueous hydrogen peroxide solution (DS9800; Blocking Peroxidase Reagent; Leica Biosystems, Germany) for 10 min. To block nonspecific binding, sections were incubated with 1% blocking reagent (11,096,176,001; Roche, Germany) diluted in phosphate-buffered saline.

Slides were then incubated at 4 °C in a humidified chamber with one of the following primary antibodies: anti-5′-methylthioadenosine phosphorylase (MTAP) (1:4,000, overnight; 11475-1-AP; Proteintech, USA), anti-MITF (1:300, overnight; D-9; Santa Cruz Biotechnology, USA), anti-CD8 (15 min; SP239; Roche, Switzerland), and anti-PD-L1 (15 min; SP263; Roche, Switzerland). For the anti-MITF antibody, a postprimary reagent (Leica Biosystems, Germany) was added for 15 min at room temperature. The next day, horseradish peroxidase-conjugated secondary antibodies (Leica Biosystems, Germany) were applied for 1 h (MTAP and MITF) or for 15 min (PD-L1 and CD8) at room temperature in a humidified chamber. Immunoreactivity was visualized using 3,3′-diaminobenzidine substrate (5 min), followed by counterstaining with hematoxylin and mounting with dibutylphthalate polystyrene xylene.

Immunohistochemistry protein expression was classified as follows: negative, + (1% to 25% positive cells), ++ (26% to 50% positive cells), and +++ (>50% positive cells). Immunostaining intensity was scored on a semiquantitative scale: 0 (negative), 1 (weak), 2 (moderate), and 3 (strong). To quantify overall marker abundance, a composite score (score = protein expression **×** immunostaining intensity) was calculated as the product of expression level and staining intensity.

### Statistical analyses

Variables following binomial distributions (i.e., response rate) were expressed as frequencies and percentages. Comparisons between qualitative variables were done using the chi-square test. Time-to-event variables (OS and PFS) were measured from the date of therapy onset and were estimated according to the Kaplan–Meier method. Comparisons between the variables of interest were performed by the log-rank test. All *P* values reported were 2-sided, and statistical significance was defined at *P* < 0.05.

## Results

**Fig. 1.**
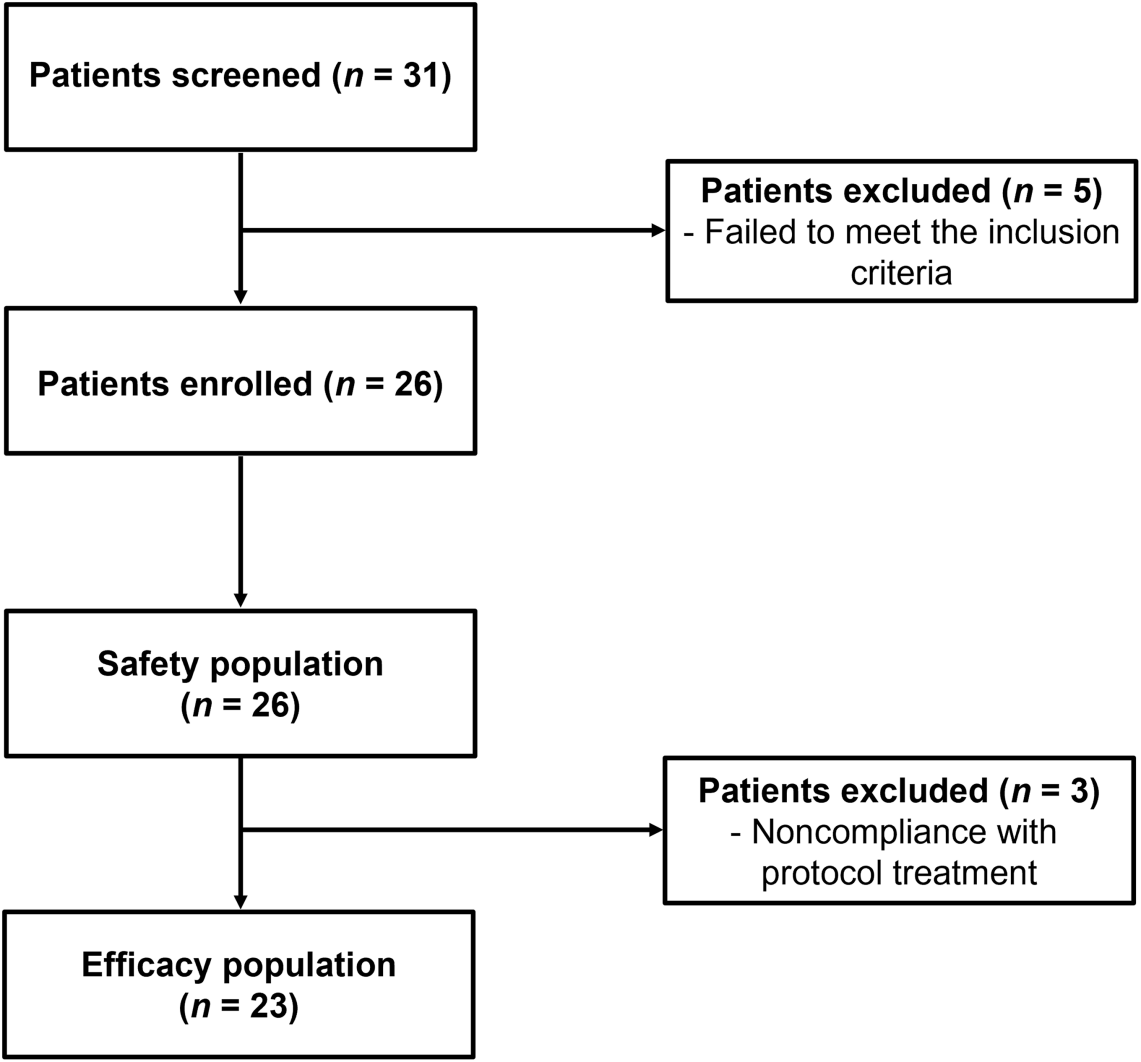
Consort diagram. The safety population consisted of patients who received at least 1 dose of the study treatment. The efficacy population consisted of patients evaluable for the main end point of the protocol.

From December 2019 to June 2024, 31 patients with advanced, progressing, and measurable CCSs were screened for eligibility. Five patients were excluded as they did not meet the inclusion and exclusion criteria, one of them having initiated the treatment. Thus, 26 patients received at least 1 dose of the study drug and constituted the safety population. Of these, 3 patients were excluded from the efficacy population due to noncompliance with the treatment established in the study protocol. However, they continued treatment until disease progression because of clinical benefits. Two patients suffered treatment interruption for several weeks within the first 3 weeks of therapy for reasons not related to the study drugs, and 1 patient received an incorrect dose of nivolumab during the first cycle. Therefore, 23 patients were evaluable for the main end point of the protocol (Fig. 1). The clinical cutoff for the final data analyses was 2025 March 3. At that time, 3 (11.5%) of the 26 patients were still on treatment, and 23 (88.5%) had discontinued treatment. Three (13.0%) of the 23 patients discontinued treatment because of toxicity: 1 because of grade 3 fatigue, 1 because of grade 3 transaminitis (alanine aminotransferase [ALT] and aspartate aminotransferase [AST] increased), and 1 because of grade 4 transaminitis (ALT increased). The remaining 20 patients (86.9%) discontinued due to progression.

A total of 193 1-month cycles of sunitinib, with a median of 5 cycles (range, 2 to 25 cycles), and 344 2-week cycles of nivolumab, with a median of 7 (range, 1 to 54 cycles), were given to the 26 patients in the safety population. A total of 15 patients (57.7%) experienced interruption of sunitinib, and 10 (38.5%) patients experienced delay of nivolumab. In 2 patients, interruptions/delays were related to the COVID-19 pandemic situation; in the remaining patients, they were due to toxicity. Sunitinib interruptions occurred at a median time of 14 weeks (range, 2 to 74 weeks) from the start of therapy, and patients could restart the drug after a median time of sunitinib interruption of 10 d (range, 1 to 55 d). In 2 patients, there were 1 and 2 subsequent sunitinib interruptions, respectively. The median delay in nivolumab administration was 12 d (range, 7 to 42 d). The median relative dose intensity for sunitinib and nivolumab was 93% (range, 17% to 100%) and 94% (range, 67% to 100%), respectively.

The primary tumor location was distributed as follows: extremities 15 (57.7%), visceral 8 (30.8%), and trunk wall 3 (11.5%). At diagnosis, 14 (53.8%) cases were localized, 4 (15.4%) were locally advanced, and 8 (30.8%) were metastatic. At study baseline, 24 (92.3%) were metastatic, and 2 (7.7%) were locally advanced. The distribution of performance status was ECOG 0 in 18 patients (69.2%) and ECOG 1 in 8 patients (30.8%). Sex distribution was balanced, with 50.0% females and 50.0% males. The median of metastasis-free interval (MFI) in this series was 4.1 months (95% confidence interval [CI], 0.0 to 238.6 months). All patients were enrolled based on a consistent morpho-immunophenotypic diagnosis and a positive fluorescence in situ hybridization result for *EWSR1* rearrangement. Sequencing data identified *EWSR1::ATF1* as the most common fusion gene product detected, with 13 (50.0%) cases, while *EWSR1::CREB1* and *EWSR1::CREM* were identified in 5 cases (19.2%) and 1 case (3.8%), respectively. Fusion gene sequencing data were not available for 7 (27.0%) patients (Table 1).

**Table 1.** Patient demographics (*n* = 26)

Characteristics	*n* (%)
Sex
Male	13 (50.0)
Female	13 (50.0)
Age at baseline, years, median (range)	42 (18–67)
Primary tumor location
Extremity	15 (57.7)
Visceral	8 (30.8)
Trunk wall	3 (11.5)
Primary tumor depth ^a^
Deep	20 (76.9)
Superficial	4 (15.4)
Information not available	2 (7.7)
Tumor extension at diagnosis
Localized	14 (53.8)
Locally advanced	4 (15.4)
Metastatic	8 (30.8)
Tumor extension at baseline
Locally advanced	2 (7.7)
Metastatic	24 (92.3)
Number of previous lines ^b^, median (range)	0 (0–4)
ECOG at baseline
0	18 (69.2)
1	8 (30.8)
MFI, months, median (range)	4.1 (0.0–238.6)
FISH for EWSR1
Positive	26 (100.0)
Negative	0 (0.0)
Fusion gene
* EWSR1::ATF1*	13 (50.0)
* EWSR1::CREB1*	5 (19.2)
* EWSR1::CREM*	1 (3.8)
Not available	7 (27.0)

*ATF1*, activating transcription factor 1; *CREB1*, CAMP-responsive element binding protein 1; *CREM*, cyclic adenosine monophosphate-responsive element modulator; ECOG, Eastern Cooperative Oncology Group; *EWSR1*, EWS RNA binding protein 1; FISH, fluorescence in situ hybridization

^a^ Superficial sarcomas are defined as tumors located entirely above the superficial fascia without fascial invasion, involving only the skin or subcutaneous tissue. Deep sarcomas are tumors located beneath the superficial fascia or those that invade or penetrate the fascia, including muscular, intermuscular, retroperitoneal, or visceral sites.

^b^ None of the patients had received previous TKI or immunotherapy. All pretreated patients (*n* = 8) had received classic cytotoxic chemotherapy.

**Fig. 2.**
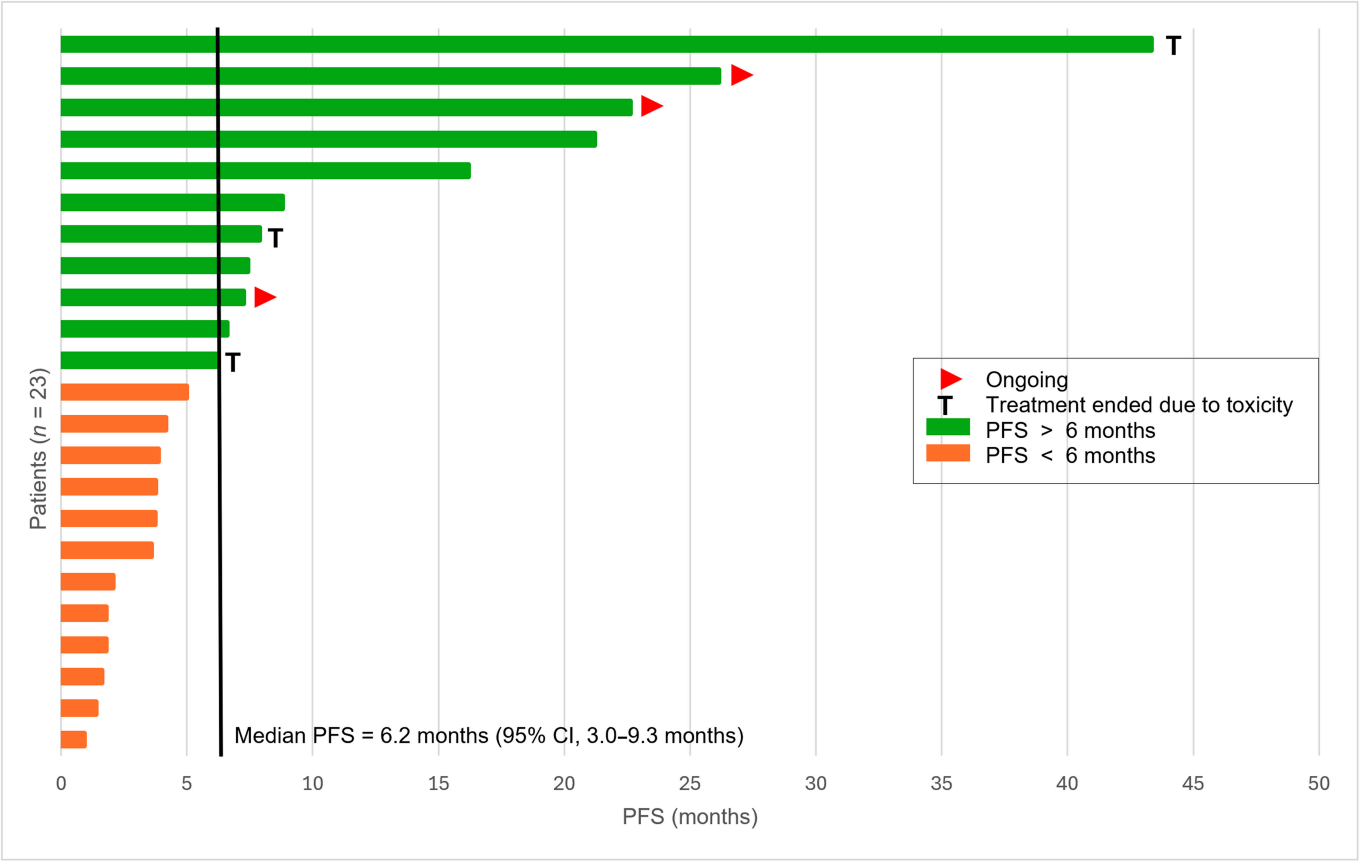
Swimmer plot illustrating PFS for patients in the efficacy population (*n* = 23). Each bar represents an individual patient from the efficacy cohort, depicting the duration of PFS from initiation of study treatment. The red arrowhead at the end of a bar indicates patients undergoing treatment at data cutoff. The symbol “T” denotes patients who discontinued therapy due to treatment-related toxicity.

With a median follow-up of 22.6 months (95% CI, 10.1 to 35.2 months), the median PFS was 6.2 months (95% CI, 3.0 to 9.3 months) (Fig. S1A), while the 6-month PFS rate was 50.1% (95% CI, 29.1 to 71.1 months) (Fig. 2). The median OS was 17.0 months (95% CI, 5.6 to 28.5 months) (Fig. S1B), and the 6- and 12-month OS rates were 73.9% (95% CI, 55.9% to 91.9%) and 56.9% (95% CI, 34.7% to 79.0%), respectively.

**Fig. 3.**
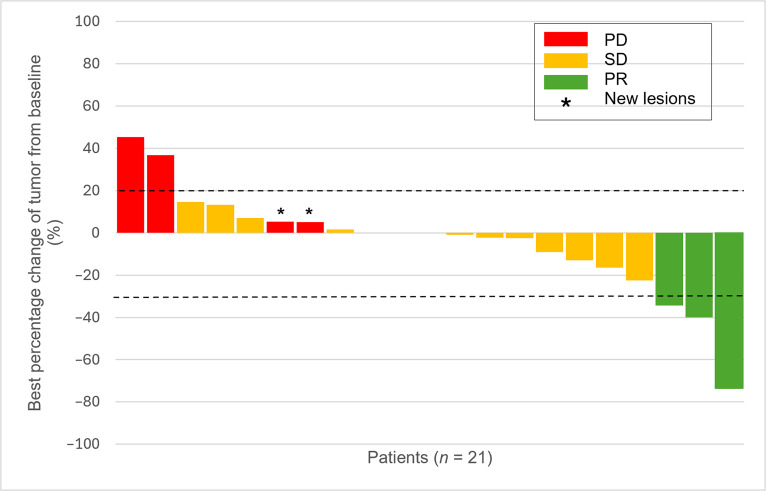
Waterfall plot of tumor response to treatment among evaluable patients (*n* = 21). Each bar represents the maximum percentage change from baseline in the sum of target lesion diameters for an individual patient, as assessed centrally by RECIST v1.1 criteria. Tumor size was measured in millimeters. The horizontal dashed lines indicate a 30% decrease and a 20% increase in tumor diameter, corresponding to thresholds for RECIST-defined PR and progressive disease (PD), respectively. Three patients did not experience changes in the sum of target lesion diameters.

Based on the central radiologic assessment, of the 23 patients evaluable for the primary end point, 21 had at least 1 radiological evaluation of response. Two patients experienced rapid clinical progression, and no adequate radiological studies were performed in these cases, although they were still evaluable for progression events. According to RECIST criteria, the best radiologic response was PR in 3 patients (14.3%), stable disease (SD) in 14 (66.7%), and progressive disease in 4 (19.0%) (Fig. 3). The median time to response was 6.5 months (95% CI, 2.7 to 10.7 months), while the median duration of response was not reached.

Hematologic adverse events were the most common toxicities in the safety population (*n* = 26), and they were distributed as follows (all grades): lymphocytopenia (46.2%), leukopenia (38.5%), anemia (38.5%), neutropenia (38.5%), thrombocytopenia (26.9%), lymphocytosis (15.4%), and leukocytosis (3.8%). Grade 5 toxicity was not observed, while grade 4 toxicities were registered in 2 of 26 patients (7.7%; 1 ALT increase and 1 ischemia). Concerning grade 3 toxicity, the distribution was as follows: lymphocytopenia (23.1%), anemia (23.1%), neutropenia (11.5%), lymphocytosis (11.5%), and 1 case each (3.8%) for fatigue, AST increase, ALT increase, hypoalbuminemia, hyponatremia, hypercalcemia, alkaline phosphatase increase, uveitis, catheter-related infection, gamma-glutamyl transferase increase, hypokalemia, hypotension, and sepsis (Table 2). Regarding nivolumab-associated immune-related adverse events, grade 1 to 2 diarrhea was observed in 5 patients (19.2%), grade 1 to 2 cutaneous rash was seen in 3 patients (11.5%), grade 1 to 2 hypothyroidism was observed in 2 patients (7.7%), grade 2 uveitis was noted in 1 patient (3.8%), and grade 3 uveitis was observed in 1 patient (3.8%). There were no immune-mediated grade 4 adverse events (Table 2). One patient discontinued sunitinib after experiencing a grade 4 thrombotic-related ischemia in a lower extremity.

**Table 2.** Toxicity profile

Item	Any grade	Grades 1–2	Grade 3	Grade 4
Hematological adverse events
Lymphocytopenia	12 (46.2%)	6 (23.1%)	6 (23.1%)	0 (0.0%)
Leukopenia	10 (38.5%)	10 (38.5%)	0 (0.0%)	0 (0.0%)
Anemia	10 (38.5%)	4 (15.4%)	6 (23.1%)	0 (0.0%)
Neutropenia	10 (38.5%)	7 (26.9%)	3 (11.5%)	0 (0.0%)
Thrombocytopenia	7 (26.9%)	7 (26.9%)	0 (0.0%)	0 (0.0%)
Lymphocytosis	4 (15.4%)	1 (3.8%)	3 (11.5%)	0 (0.0%)
Leukocytosis	1 (3.8%)	1 (3.8%)	0 (0.0%)	0 (0.0%)
Nonhematological adverse events
Fatigue	9 (34.6%)	8 (30.8%)	1 (3.8%)	0 (0.0%)
AST increased	8 (30.8%)	7 (26.9%)	1 (3.8%)	0 (0.0%)
Skin and SC tissue disorders ^a^	8 (30.8%)	8 (30.8%)	0 (0.0%)	0 (0.0%)
ALT increased	7 (26.9%)	5 (19.2%)	1 (3.8%)	1 (3.8%)
Diarrhea ^a^	6 (23.1%)	6 (23.1%)	0 (0.0%)	0 (0.0%)
Dysgeusia	5 (19.2%)	5 (19.2%)	0 (0.0%)	0 (0.0%)
Mucositis oral	5 (19.2%)	5 (19.2%)	0 (0.0%)	0 (0.0%)
Nausea	5 (19.2%)	5 (19.2%)	0 (0.0%)	0 (0.0%)
Hypoalbuminemia	4 (15.4%)	3 (11.5%)	1 (3.8%)	0 (0.0%)
Anorexia	3 (11.5%)	3 (11.5%)	0 (0.0%)	0 (0.0%)
Hyperkalemia	3 (11.5%)	3 (11.5%)	0 (0.0%)	0 (0.0%)
Hypertension	3 (11.5%)	3 (11.5%)	0 (0.0%)	0 (0.0%)
Hypocalcemia	3 (11.5%)	3 (11.5%)	0 (0.0%)	0 (0.0%)
Hyponatremia	3 (11.5%)	2 (7.7%)	1 (3.8%)	0 (0.0%)
Pain	3 (11.5%)	3 (11.5%)	0 (0.0%)	0 (0.0%)
Creatinine increased	2 (7.7%)	2 (7.7%)	0 (0.0%)	0 (0.0%)
Hypercalcemia	2 (7.7%)	1 (3.8%)	1 (3.8%)	0 (0.0%)
ALP increased	2 (7.7%)	1 (3.8%)	1 (3.8%)	0 (0.0%)
Blood lactate dehydrogenase increased	2 (7.7%)	2 (7.7%)	0 (0.0%)	0 (0.0%)
CPK increased	2 (7.7%)	2 (7.7%)	0 (0.0%)	0 (0.0%)
Epistaxis	2 (7.7%)	2 (7.7%)	0 (0.0%)	0 (0.0%)
Headache	2 (7.7%)	2 (7.7%)	0 (0.0%)	0 (0.0%)
Hypothyroidism ^a^	2 (7.7%)	2 (7.7%)	0 (0.0%)	0 (0.0%)
Oral hemorrhage	2 (7.7%)	2 (7.7%)	0 (0.0%)	0 (0.0%)
Palmar–plantar erythrodysesthesia syndrome	2 (7.7%)	2 (7.7%)	0 (0.0%)	0 (0.0%)
Uveitis ^a^	2 (7.7%)	1 (3.8%)	1 (3.8%)	0 (0.0%)
Vomiting	2 (7.7%)	2 (7.7%)	0 (0.0%)	0 (0.0%)
Alopecia	1 (3.8%)	1 (3.8%)	0 (0.0%)	0 (0.0%)
Amylase increased	1 (3.8%)	1 (3.8%)	0 (0.0%)	0 (0.0%)
Catheter-related infection	1 (3.8%)	0 (0.0%)	1 (3.8%)	0 (0.0%)
Cholesterol high	1 (3.8%)	1 (3.8%)	0 (0.0%)	0 (0.0%)
Cyanosis	1 (3.8%)	1 (3.8%)	0 (0.0%)	0 (0.0%)
Dry mouth	1 (3.8%)	1 (3.8%)	0 (0.0%)	0 (0.0%)
Dyspepsia	1 (3.8%)	1 (3.8%)	0 (0.0%)	0 (0.0%)
Edema limbs	1 (3.8%)	1 (3.8%)	0 (0.0%)	0 (0.0%)
Ejection fraction decreased	1 (3.8%)	1 (3.8%)	0 (0.0%)	0 (0.0%)
Fever	1 (3.8%)	1 (3.8%)	0 (0.0%)	0 (0.0%)
GGT increased	1 (3.8%)	0 (0.0%)	1 (3.8%)	0 (0.0%)
Gastroesophageal reflux disease	1 (3.8%)	1 (3.8%)	0 (0.0%)	0 (0.0%)
Hemorrhoidal hemorrhage	1 (3.8%)	1 (3.8%)	0 (0.0%)	0 (0.0%)
Hyperglycemia	1 (3.8%)	1 (3.8%)	0 (0.0%)	0 (0.0%)
Hyperthyroidism	1 (3.8%)	1 (3.8%)	0 (0.0%)	0 (0.0%)
Hypokalemia	1 (3.8%)	0 (0.0%)	1 (3.8%)	0 (0.0%)
Hypomagnesemia	1 (3.8%)	1 (3.8%)	0 (0.0%)	0 (0.0%)
Hypophosphatemia	1 (3.8%)	1 (3.8%)	0 (0.0%)	0 (0.0%)
Hypotension	1 (3.8%)	0 (0.0%)	1 (3.8%)	0 (0.0%)
Lipase increased	1 (3.8%)	1 (3.8%)	0 (0.0%)	0 (0.0%)
Muscle weakness upper limb	1 (3.8%)	1 (3.8%)	0 (0.0%)	0 (0.0%)
Myalgia	1 (3.8%)	1 (3.8%)	0 (0.0%)	0 (0.0%)
Myocarditis	1 (3.8%)	1 (3.8%)	0 (0.0%)	0 (0.0%)
Paresthesia	1 (3.8%)	1 (3.8%)	0 (0.0%)	0 (0.0%)
Proteinuria	1 (3.8%)	1 (3.8%)	0 (0.0%)	0 (0.0%)
Sepsis	1 (3.8%)	0 (0.0%)	1 (3.8%)	0 (0.0%)
Somnolence	1 (3.8%)	1 (3.8%)	0 (0.0%)	0 (0.0%)
Thyroid-stimulating hormone increased	1 (3.8%)	1 (3.8%)	0 (0.0%)	0 (0.0%)
Urine discoloration	1 (3.8%)	1 (3.8%)	0 (0.0%)	0 (0.0%)
Ischemia	1 (3.8%)	0 (0.0%)	0 (0.0%)	1 (3.8%)
Weight gain	1 (3.8%)	1 (3.8%)	0 (0.0%)	0 (0.0%)

ALP, alkaline phosphatase; ALT, alanine aminotransferase; AST, aspartate aminotransferase; CPK, creatine phosphokinase; GGT, gamma-glutamyl transferase; SC, subcutaneous

^a^ Immune-related adverse events (irAEs): diarrhea in 5 patients (19.2%), grade 1 to 2 cutaneous rash in 3 patients (11.5%), grade 1 to 2 hypothyroidism in 2 patients (7.7%), grade 2 uveitis in 1 patient (3.8%), and grade 3 uveitis in 1 patient (3.8%).

In exploratory univariate Cox regression analyses, ECOG 1 was associated with a significantly worse PFS compared to ECOG 0 (hazard ratio = 2.7 [95% CI, 1.0 to 6.9]; *P* = 0.046). Meanwhile, the different fusion gene partners or the number of previous therapy lines did not have an impact on survival (Table S2). In the correlative studies, PD-L1 expression was positive in 65.4% (17/26) of the cases, whereas CD8 infiltration was detected in 76.9% (20/26) of the CCSs. Moreover, MTAP and MITF expression in >50% of tumor cells was observed in 21 of 26 cases (80.8%) and 20 of 26 cases (76.9%), respectively. The complete distribution of protein expression levels and staining intensities for the different antibodies is detailed in Table S3. Higher PD-L1 composite score (>3) was associated with longer median PFS (21.2 [95% CI, 6.0 to 36.4] months versus 4.2 [95% CI, 2.7 to 5.6] months, *P* = 0.045) (Fig. S2 and Table S4). Examples of PD-L1 protein expression are shown in Fig. S3. CD8 did not show statistically significant associations with survival (Table S4). Of note, a high PD-L1 composite score also correlated significantly with response (*P* = 0.011) (Table S5).

## Discussion

The combination of sunitinib and nivolumab in the CCS cohort of the IMMUNOSARC phase II study showed a clinically meaningful activity, achieving a 6-month PFS rate of 50.1% (11 patients progression-free at 6 months) and a promising median OS of 17.0 months. The results of our trial compare favorably with those of previous prospective trials (Table 3). For instance, in the tivantinib trial, the CCS cohort (*n* = 11) showed a 6-month PFS rate of 21% and a median OS (mOS) of 5.2 months [5], while in the crizotinib trial, among 28 patients with CCS, the 6-month PFS rate and mOS were 27% and 9 months, respectively [6]. These findings suggest that the sunitinib and nivolumab combination may offer more than double the PFS and OS compared to MET inhibitors, based on indirect comparisons. Of interest, these encouraging results were seen in a particularly aggressive setting, as indicated by a median MFI of only 4.1 months, an established key prognostic factor for PFS in advanced soft-tissue sarcoma (STS) [11].

**Table 3.** Prospective available evidence on tyrosine kinase inhibitors and PD-1/PD-L1 inhibitors in clear cell sarcoma

Regimen	*n*	ORR (RECIST), *n* (%)	mPFS (months)	6-month PFS (%)	mOS (months)	Reference
Tivantinib	11	1 (9.1)	1.9	21.0	5.2	[5]
Crizotinib	28	1 (3.6)	4.3	27.0	7.8	[6]
Pazopanib	1	0 (0.0)	10.3	100.0	23.2	[16]
Nivolumab	11	0 (0.0)	4.1	25.0	13.2	[33]
Pembrolizumab	3	0 (0.0)	2.1^a^	NA	7.0 ^a^	[34]
Sunitinib plus nivolumab	11	2 (18.2)	5.2	NA	8.4	[7]
Anlotinib plus sintilimab	3	1 (33.3)	NA	NA	NA	[35]
Sunitinib plus nivolumab	21	3 (14.3)	6.2	50.1	17.0	This study

ORR, objective response rate; mOS, median overall survival; mPFS, median progression-free survival; NA, not available; PD-1, programmed cell death protein 1; PD-L1, programmed death-ligand 1; RECIST, response evaluation criteria in solid tumors

^a^ Data for the “other sarcoma” cohort included 3 clear cell sarcoma cases.

Other TKIs have been tested in the setting of advanced CCS and reported in retrospective series or as case reports. An international effort reported 3 PRs out of 10 patients diagnosed with advanced CCS after sunitinib treatment, with a median OS of 15 months (95% CI, 3 to 27 months). However, the median PFS was just 4 months (95% CI, 1 to 7 months) [3]. Sunitinib had previously shown clinical activity in CCS [12] as well as the modulation of the tumor microenvironment toward a higher inflamed state, with an accumulation of CD3^+^ and CD8^+^ lymphocytes in the post-sunitinib tumor specimen [13]. Additionally, sorafenib and pazopanib have also been linked to responses [14,15] or prolonged disease control [16,17]. Some authors hypothesize that this activity could be related to vascular endothelial growth factor receptor inhibition. Actually, the underlying chimeric EWSR1–ATF1 protein targets the hypoxia-inducible factor-1α through MITF. This triggers the expression of proteins critical for neoangiogenesis, including vascular endothelial growth factor A. The activity of antiangiogenics detected in CCS could be consistent with the inhibition of this pathway. In our series, MITF was expressed in almost all the cases, confirming that this protein is a likely target of the *EWSR1*-fusion gene driver of the disease, as previously suggested [4]. However, it is still unclear whether the mechanism is by facilitating adaptive immune response through neoangiogenesis inhibition [8].

The fact that CCS exhibits melanocytic differentiation gene products such as *MITF* and shares mRNA expression patterns with melanoma, including melanocyte differentiation antigens, the SRY-box transcription factor 10 (*SOX10*), and the growth factor receptors erb-b2 receptor tyrosine kinase 3 (*ERBB3*) and fibroblast growth factor receptor 1 (*FGFR1*) [18], has prompted consideration of immunomodulation in CCS, mirroring its significance in melanoma. Along this line of thought, a phase I trial was conducted using autologous granulocyte-macrophage colony-stimulating factor stemming from CCS metastatic lesions after transduction with an adenoviral vector encoding this cytokine. A minimum of 6 immunizations were administered per patient intradermally and subcutaneously. Of the 3 patients diagnosed with CCS enrolled in this trial, 1 died of progression at 4 months, 1 died of disease at 24 months, and another patient is alive 103 months later, counting from treatment initiation [19]. The combination of cabozantinib with PD-1 inhibitor nivolumab and an autologous tumor cell vaccine achieved a PFS of 2 years in a 9-year-old patient diagnosed with an aggressive CCS [20]. Real-life data from a Chinese population analyzed the combination of immunotherapy plus antiangiogenics in STS. They reported a median PFS of 16.5 months (95% CI, 0.0 to 35.9 months) in 5 registered patients diagnosed with CCS [21].

Our previous phase I/II trial (IMMUNOSARC I) obtained 2 PRs out of 11 (18.2%) subjects diagnosed with CCS with the combination of sunitinib plus nivolumab [7], which is consistent with the 14.3% found in the current cohort of the IMMUNOSARC II trial. In the waterfall plot, 10 of 21 (47.6%) patients had some tumor shrinkage. Interestingly, the median duration of response of the 3 responders was not reached. One patient progressed after 14.1 months, while the remaining patients were still without progression after 22.6 and 26.1 months. These prolonged responses are more typically related to immunomodulation. Moreover, 2 patients who achieved SD as the best response had long-lasting stabilizations of 45.6 and 21.2 months, respectively. Another patient with SD is under treatment with no progression after 7.3 months. With regard to classic cytotoxic chemotherapy, retrospective data reviewing the impact of chemotherapy in advanced CCS coincide with the observation that doxorubicin monotherapy alone does not show meaningful clinical activity in CCS. In a series of 24 individuals with advanced CCS, 1 patient (4%) reached PR following doxorubicin and ifosfamide, 9 (38%) had SD, and 14 (58%) had disease progression [22]. Other authors reported a PR rate of 23.3% (7/30) among patients with measurable advanced CCS. Notably, all 7 responders had received a doxorubicin–cisplatin-based combination (5 received doxorubicin plus cisplatin, and 2 received doxorubicin, cisplatin, and ifosfamide) [23]. In contrast, none of the patients treated with doxorubicin monotherapy in either series achieved an objective response.

Our findings suggest that PD-L1 expression, captured by a composite score combining expression level and staining intensity, may serve as a predictive biomarker for the activity of anti-PD-1 inhibitors in CCS. This observation contrasts with previous studies conducted across broader or unselected STS populations, where PD-L1 expression did not predict clinical benefit from immune checkpoint inhibitors, including anti-PD-1 agents such as pembrolizumab [24]. Moreover, in our series, 66% of CCSs exhibited positive immunostaining for PD-L1, a value notably higher than those reported in previous studies of unselected STS [25,26]. Of note, diffuse overexpression of PD-L1 has been reported in angiomatoid fibrous histiocytoma, another tumor type that can harbor the *EWSR1*-fusion genes with *CREB1* or *ATF1* [27]. ATF1 and CREB1 share several functional and structural properties, as both belong to the CREB/ATF family of basic leucine zipper transcription factors. Both bind to the consensus CRE sequence in promoters of target genes, and these results suggest that the EWSR1 fusions may regulate PD-L1 expression through mechanisms yet to be explored. A potential hypothesis would be related to the fact that fusion genes like *EWSR1::ATF1* can generate novel protein sequences at the fusion break point, which are processed and presented as neoantigens by tumor cells via human leukocyte antigen class I molecules [28]. These neoantigens are recognized by the immune system, potentially triggering an antitumor immune response. When the immune system detects these neoantigens, tumor-infiltrating T cells become activated, producing cytokines such as interferon-γ, which is a potent inducer of PD-L1 expression [29]. Overexpression of PD-L1 may help tumor cells evade the antitumor immune response. To our knowledge, this is one of the first studies to demonstrate a clinically meaningful association between PD-L1 expression—quantified using a composite scoring approach—and outcomes in an ultrarare sarcoma subtype. These findings suggest that qualitative and quantitative features of PD-L1 expression, beyond simple positivity, may be particularly relevant in biologically homogeneous, fusion-driven tumors such as CCS. Future prospective trials should consider incorporating standardized PD-L1 composite scoring methodologies, including predefined thresholds and centralized pathology review, to improve reproducibility across studies. Nonetheless, PD-L1 remains an imperfect biomarker, limited by spatial and temporal heterogeneity, assay variability, and the need for prospective validation, particularly in ultrarare tumor populations.

On the other hand, protein arginine methyltransferase 5 (PRMT5) has been reported to play a critical role in mediating gene transcription driven by the *EWSR1::ATF1* oncogenic fusion in CCS [30]. Additionally, MTAP-deficient tumors are known to exhibit increased dependency on PRMT5 expression and activity for proliferation, with *MTAP* loss creating a synthetic vulnerability that can be therapeutically exploited [31]. However, in our cohort, MTAP expression was preserved in the majority of cases, suggesting that the potential efficacy of PRMT5 inhibition in CCS may be more likely attributable to its essential role in oncogenic transcriptional regulation rather than to a synthetic lethal interaction resulting from MTAP loss.

The absence of paired tumor samples in this phase II cohort represents a study limitation, as it precludes evaluation of treatment-induced dynamic molecular alterations. Furthermore, budgetary constraints restricted the extent of correlative analyses, such as transcriptomic profiling. An additional limitation is the small sample size inherent to studies conducted in ultrarare tumors such as CCS, which limits statistical power and the generalizability of the findings. Moreover, the single-arm, nonrandomized design precludes definitive conclusions regarding the relative contribution of nivolumab to the observed clinical activity, and it remains unclear whether the addition of immune checkpoint blockade enhanced the known activity of sunitinib or other angiogenesis inhibitors. Nevertheless, targeting immunogenic cell death has recently shown promising data of activity in some advanced STS [32]. Considering that platinum compounds are inducers of immunogenic cell death, and they have exhibited intriguing activity in CCS, it seems logical to prospectively test the combination of platinum plus immune checkpoint inhibitors in this context.

## Conclusions

The results of this trial indicate that the combination of sunitinib 25 mg/d (after an induction phase of 2 weeks at 37.5 mg/d) plus nivolumab 240 mg every 2 weeks demonstrates clinical activity in advanced CCS. Outcomes such as a 6-month PFS rate of 50.1%, an ORR of 14.3%, and long-term responders, with 5 (21.7%) patients experiencing no disease progression at 1 year and 4 remaining progression-free at 20 months, among those achieving response or stabilization, are encouraging. Unfortunately, neither of these drugs is approved for use in STS, including CCS. The hope is that mechanisms such as drug repurposing, with the support of the sarcoma community (including the sarcoma patient advocacy group), may allow access to them in the future. This study provides a benchmark for clinical activity in patients with advanced CCS and supports the hypothesis that immunomodulation is relevant in this disease.

## Ethical Approval

Written authorization from the patient or legal guardians was required before the initiation of study-specific procedures. All procedures adhered to the standards outlined by each hospital’s ethics committee and followed the principles articulated in the Declaration of Helsinki. Ethical approval was obtained from the ethics committee at each participating institution (Table S1).

## Data Availability

The clinical dataset, with anonymized participant data regarding demographics, treatment information, and outcomes, will be available upon request to the corresponding author for related research and following the signature of a specific agreement to this end.
