# Supplementary material for: IMMUNOSARC II Master Trial: Phase II Study of Sunitinib and Nivolumab in Clear Cell Sarcoma Cohort
Source: Cancer Commun (Lond). 2026 Mar 12;46:0015. doi: 10.34133/cancomm.0015 (PMC12981246; doi:10.34133/cancomm.0015)
Supplement: Supplementary 1 — Tables S1 to S5 Figs. S1 to S3 Supplementary Text [file cancomm.0015.f1.zip › CANCOMM-D-25-00140-Supplementary Materials-Final.docx]

**Supplementary Materials**

**IMMUNOSARC II master trial: phase II study of sunitinib and nivolumab in clear cell sarcoma cohort**

Running title: Sunitinib and nivolumab in clear cell sarcoma

Javier Martin-Broto^1, 2, 3, *^, Sandra J. Strauss^4^, Emanuela Palmerini^5,6^, Claudia Valverde^7^, Ana Sebio^8^, Andres Redondo^9^, Silvia Stacchiotti^10^, Giovanni Grignani^11^, Sandra Aliberti^11^, Roberto Diaz-Beveridge^12^, Enrique Gonzalez Billalabeitia^13^, Josefina Cruz^14^, Irene Carrasco-Garcia^15^, Toni Ibrahim^5^, Juan Diaz-Martin^16, 17^, Carmen Salguero-Aranda^16, 17^, Antonio Gutierrez^18^, Empar Mayordomo-Aranda^19^, Rafael Ramos^20^, Jose Merino^21^, Paola Collini^22^, Roberto Tirabosco^23^, Silvia Bague^24^, Cleofe Romagosa^25^, Maria Augusta Carrera^2, 3^, Patricio Ledesma^26^, Nadia Hindi^1, 2, 3^, and David Silva Moura^2, 3^

^1^ Department of Medical Oncology, Fundación Jiménez Díaz University Hospital, Madrid, Spain;

^2^ University Hospital General de Villalba, Madrid, Spain;

^3^ Research health institute of Fundación Jiménez Díaz, Autonomous university of Madrid, Madrid, Spain;

^4^ Department of Medical Oncology, University College London Hospital, London, United Kingdom;

^5^ Department of Medical Oncology, Istituto di Ricovero e Cura a Carattere Scientifico, Istituto Ortopedico Rizzoli, Bologna, Italy;

^6^ Department of Medical Oncology, Sylvester Comprehensive Cancer Center, Miller School of Medicine, University of Miami, Miami, Florida, USA;

^7^ Department of Medical Oncology, University Hospital Vall d’Hebron, Barcelona, Spain;

^8^ Department of Medical Oncology, Sant Pau Hospital, Barcelona, Spain;

^9^ Department of Medical Oncology, University Hospital La Paz, Madrid, Spain;

^10^ Medical Oncology Department, Fondazione Istituto di Ricovero e Cura a Carattere Scientifico, Istituto Nazionale dei Tumori, Milan, Italy;

^11^ Medical Oncology Department, Candiolo Cancer Institute, Fondazione Piemontese per l’Oncologia-Istituto di Ricovero e Cura a Carattere Scientifico (FPO-IRCCS), Candiolo, Italy;

^12^ Department of Medical Oncology, University Hospital La Fe, Valencia, Spain;

^13^ Department of Medical Oncology, University Hospital 12 de Octubre, Madrid, Spain;

^14^ Department of Medical Oncology, Canarias University Hospital, La Laguna, Spain;

^15^ Department of Medical Oncology, University Hospital Virgen del Rocio, Seville, Spain;

^16^ Institute of Biomedicine of Seville, Spanish National Research Council-University of Seville, Department of Pathology, University Hospital Virgen del Rocio, Seville, Spain;

^17^ Biomedical Research Networking Center in Cancer (CIBERONC), Carlos III Health Institute, Madrid, Spain;

^18^ Department of Hematology, University Hospital Son Espases, Palma de Mallorca, Spain;

^19^ Department of Pathology, University Hospital La Fe, Valencia, Spain;

^20^ Department of Pathology, University Hospital Son Espases, Palma de Mallorca, Spain;

^21^ Department of Pathology, Fundación Jiménez Díaz University Hospital, Madrid, Spain;

^22^ Advanced Diagnostic Department, Fondazione Istituto di Ricovero e Cura a Carattere Scientifico Istituto Nazionale dei Tumori, Milan, Italy;

^23^ Department of Pathology, University College London Hospital, London, United Kingdom;

^24^ Department of Pathology, Sant Pau Hospital, Barcelona, Spain;

^25^ Department of Pathology, University Hospital Vall d’Hebron, Barcelona, Spain;

^26^ Sofpromed Investigación Clínica, Palma de Mallorca, Spain.

**^*^Corresponding Author:**

Dr. Javier Martin-Broto, Department of Medical Oncology, Fundacion Jimenez Diaz University Hospitals, Madrid, 28040 Madrid, Spain, jmartin@atbsarc.org

**Supplementary Table S1. Number of patients recruited by each participating center.**

| Country | Site | Patients recruited | Ethics approval code |
| --- | --- | --- | --- |
| Spain | Hospital de la Santa Creu i Sant Pau | 3 | GEIS-52 |
| Spain | Hospital Universitario Vall d'Hebron | 3 | GEIS-52 |
| Spain | Hospital Universitario Fundación Jiménez Díaz | 1 | GEIS-52 |
| Spain | Hospital Universitario La Paz | 2 | GEIS-52 |
| Spain | Hospital Clínico de San Carlos | 1 | GEIS-52 |
| Spain | Hospital Universitario 12 de Octubre | 1 | GEIS-52 |
| Spain | Hospital Universitario de Canarias | 1 | GEIS-52 |
| Spain | Hospital Universitario Virgen del Rocío | 1 | GEIS-52 |
| Spain | Hospital Universitario y Politécnico de La Fe | 1 | GEIS-52 |
| UK | University College Hospital | 5 | 20/LO/0978 |
| Italy | Istituto Ortopedico Rizzoli | 4 | 209/2018/Farm/IOR_EM1 |
| Italy | Istituto Nazionale dei Tumori | 2 | INT 210/17 |
| Italy | Fondazione del Piemonte per l'Oncologia | 1 | Prot. 27 del 21 gennaio 2021 |

**Supplementary Table S2. Univariate Cox regression analysis.**

| **Variables** | ***n*** | **PFS**  **HR (95% CI)** | ***P*** | **OS**  **HR (95%CI)** | ***P*** |
| --- | --- | --- | --- | --- | --- |
| Sex | | | 0.405 | 0.479 | |
| Male | 13 | Reference |  | Reference |  |
| Female | 10 | 0.7 (0.3-1.7) |  | 0.7 (0.2-2.0) |  |
| Age, years | | | 0.592 | 0.882 | |
| 18-42 | 10 | Reference |  | Reference |  |
| >42 | 13 | 1.3 (0.5-3.3) |  | 0.9 (0.3-2.6) |  |
| Primary tumor location | | | 0.127 | 0.448 | |
| Extremity | 14 | Reference |  | Reference |  |
| Visceral | 7 | 0.3 (0.1-1.0) |  | 0.5 (0.1-1.9) |  |
| Trunk wall | 2 | 0.7 (0.1-3.1) |  | 1.5 (0.3-7.3) |  |
| Primary tumor depth^a^ | | | 0.578 | 0.146 | |
| Superficial |  | Reference |  | Reference |  |
| Deep | 18 | 1.4 (0.4-4.9) |  | 4.6 (0.6-36.1) |  |
| Tumor status at diagnosis | | | 0.674 | 0.542 | |
| Localized | 13 | Reference |  | Reference |  |
| Locally advanced | 2 | 1.0 (0.2-4.8) |  | 2.1 (0.4-11.4) |  |
| Metastatic | 8 | 0.6 (0.2-1.8) |  | 1.8 (0.5-5.9) |  |
| Tumor status at baseline | | | 0.358 | 0.658 | |
| Locally advanced | 2 | Reference |  | Reference |  |
| Metastatic | 21 | 2.6 (0.3-19.5) |  | 1.6 (0.2-12.3) |  |
| Previous lines | | | 0.186 | 0.223 | |
| 0 | 17 | Reference |  | Reference |  |
| 1 or more | 6 | 0.5 (0.1-1.4) |  | 0.4 (0.1-1.7) |  |
| ECOG baseline | | | 0.046 | 0.125 | |
| 0 | 16 | Reference |  | Reference |  |
| 1 | 7 | 2.7 (1.0-6.9) |  | 2.5 (0.8-7.9) |  |
| Gene fusion | | | 0.361 | 0.999 | |
| *EWSR1::ATF1* | 11 | Reference |  | Reference |  |
| *EWSR1::CREB1* | 5 | 3.9 (0.4-38.2) |  | 1.0 (0.1-11.6) |  |
| *EWSR1::CREM* | 1 | NA |  | NA |  |

^a^Superficial sarcomas are defined as tumors located entirely above the superficial fascia without fascial invasion, involving only the skin or subcutaneous tissue. Deep sarcomas are tumors located beneath the superficial fascia or those that invade or penetrate the fascia, including muscular, intermuscular, retroperitoneal, or visceral sites. Abbreviations: *ATF1*, Activating transcription factor 1; CI, Confidence interval; *CREB1*, CAMP-responsive element binding protein 1; *CREM*, cAMP-responsive element modulator; ECOG, Eastern Cooperative Oncology Group; *EWSR1*, Ewing Sarcoma breakpoint region 1; NA, Not available; PFS, Progression-free survival; OS, Overall survival.

**Supplementary Table S3. Protein expression analysis.**

| Variables | Evaluable for toxicity (*n* = 26) | Evaluable for response  (*n* = 21) | Evaluable for survival  (*n* = 23) |
| --- | --- | --- | --- |
| CD8 expression | | | |
| Negative | 2 (7.7%) | 2 (9.5%) | 2 (8.7%) |
| + | 18 (69.3%) | 13 (61.9%) | 15 (65.3%) |
| ++ | 1 (3.8%) | 1 (4.8%) | 1 (4.3%) |
| +++ | 1 (3.8%) | 1 (4.8%) | 1 (4.3%) |
| Not available | 4 (15.4%) | 4 (19.0%) | 4 (17.4%) |
| CD8 intensity | | | |
| 0 | 2 (7.7%) | 2 (9.5%) | 2 (8.7%) |
| 1 | 0 (0.0%) | 0 (0.0%) | 0 (0.0%) |
| 2 | 0 (0.0%) | 0 (0.0%) | 0 (0.0%) |
| 3 | 20 (76.9%) | 15 (71.5%) | 17 (73.9%) |
| Not available | 4 (15.4%) | 4 (19.0%) | 4 (17.4%) |
| PD-L1 expression | | | |
| Negative | 5 (19.2%) | 4 (19.0%) | 4 (17.4%) |
| + | 11 (42.3%) | 8 (38.2%) | 10 (43.5%) |
| ++ | 4 (15.4%) | 4 (19.0%) | 4 (17.4%) |
| +++ | 2 (7.7%) | 1 (4.8%) | 1 (4.3%) |
| Not available | 4 (15.4%) | 4 (19.0%) | 4 (17.4%) |
| PD-L1 intensity | | | |
| 0 | 5 (19.2%) | 4 (19.0%) | 4 (17.4%) |
| 1 | 1 (3.8%) | 1 (4.8%) | 1 (4.3%) |
| 2 | 7 (27.0%) | 6 (28.6%) | 6 (26.1%) |
| 3 | 9 (34.6%) | 6 (28.6%) | 8 (34.8%) |
| Not available | 4 (15.4%) | 4 (19.0%) | 4 (17.4%) |
| MTAP expression | | | |
| Negative | 0 (0.0%) | 0 (0.0%) | 0 (0.0%) |
| + | 1 (3.8%) | 0 (0.0%) | 1 (4.3%) |
| ++ | 0 (0.0%) | 0 (0.0%) | 0 (0.0%) |
| +++ | 21 (80.8%) | 17 (81.0%) | 18 (78.3%) |
| Not available | 4 (15.4%) | 4 (19.0%) | 4 (17.4%) |
| MTAP intensity | | | |
| 0 | 0 (0.0%) | 0 (0.0%) | 0 (0.0%) |
| 1 | 1 (3.8%) | 0 (0.0%) | 1 (4.3%) |
| 2 | 2 (7.7%) | 2 (9.5%) | 2 (8.7%) |
| 3 | 19 (73%) | 15 (71.5%) | 16 (69.6%) |
| Not available | 4 (15.4%) | 4 (19.0%) | 4 (17.4%) |
| MITF expression | | | |
| Negative | 1 (3.8%) | 1 (4.8%) | 1 (4.3%) |
| + | 0 (0.0%) | 0 (0.0%) | 0 (0.0%) |
| ++ | 1 (3.8%) | 1 (4.8%) | 1 (4.3%) |
| +++ | 20 (76.9%) | 15 (71.5%) | 17 (73.9%) |
| Not available | 4 (15.4%) | 4 (19.0%) | 4 (17.4%) |
| MITF intensity | | | |
| 0 | 1 (3.8%) | 1 (4.8%) | 1 (4.3%) |
| 1 | 0 (0.0%) | 0 (0.0%) | 0 (0.0%) |
| 2 | 0 (0.0%) | 0 (0.0%) | 0 (0.0%) |
| 3 | 21 (80.8%) | 16 (76.2%) | 18 (78.3%) |
| Not available | 4 (15.4%) | 4 (19.0%) | 4 (17.4%) |

Abbreviations: MITF, melanocyte inducing transcription factor; MTAP, methylthioadenosine phosphorylase; PD-L1, programmed death-ligand 1. Immunohistochemistry protein expression was classified as follows: negative, + (1%–25% positive cells), ++ (26%–50% positive cells), and +++ (>50% positive cells). Immunostaining intensity was scored on a semi-quantitative scale: 0 (negative), 1 (weak), 2 (moderate), and 3 (strong).


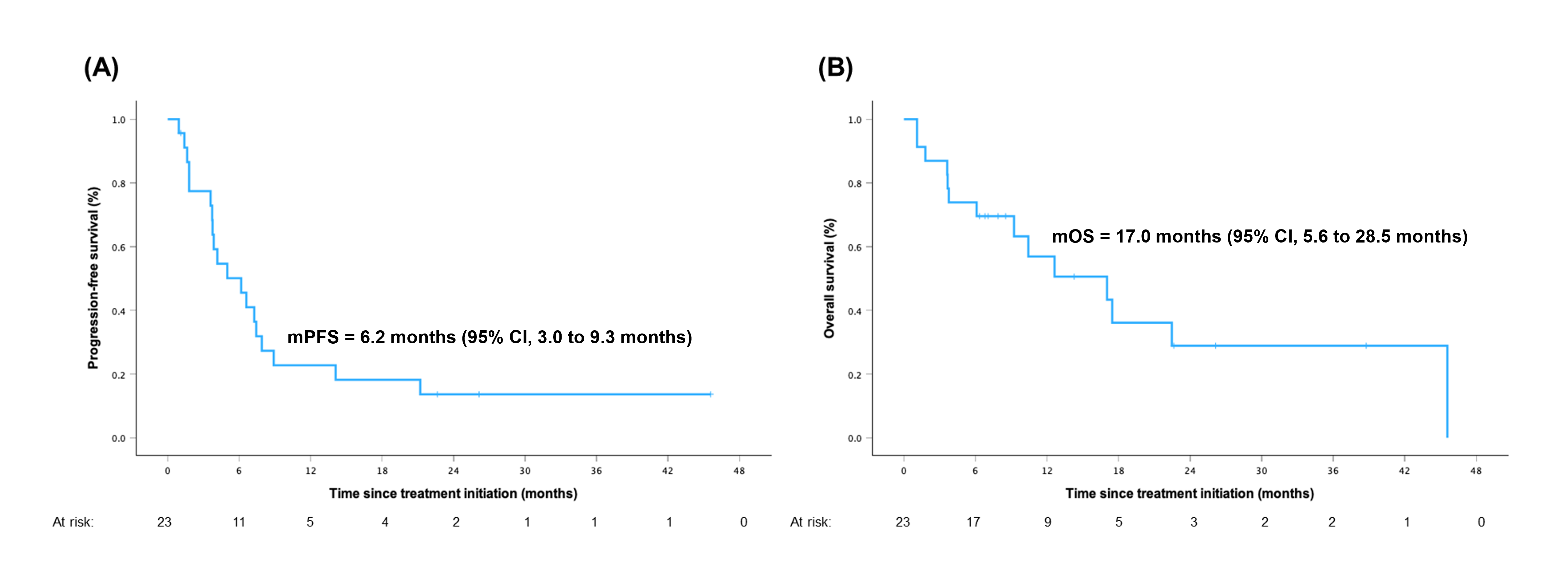


**Supplementary Figure S1. Survival analyses.** Kaplan-Meier curves for (A) progression-free survival (PFS) and (B) overall survival (OS). Abbreviations: CI, confidence interval; mPFS, median progression-free survival; mOS, median overall survival.

**
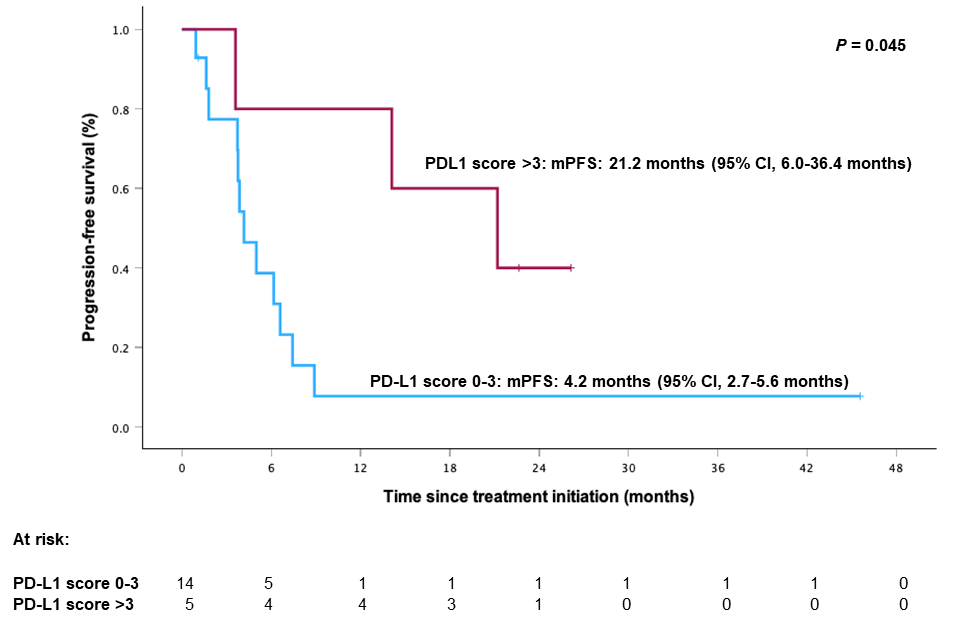
**

**Supplementary Figure S2. Predictive value of PD-L1 protein expression.** The composite score was calculated as the product of expression level and staining intensity. Patients with a score higher than 3 points were compared with patients with a score between 0 and 3. Survival curves were compared using the log-rank test. Abbreviations: CI, confidence interval; mPFS, median progression-free survival; PD-L1, programmed death-ligand 1.


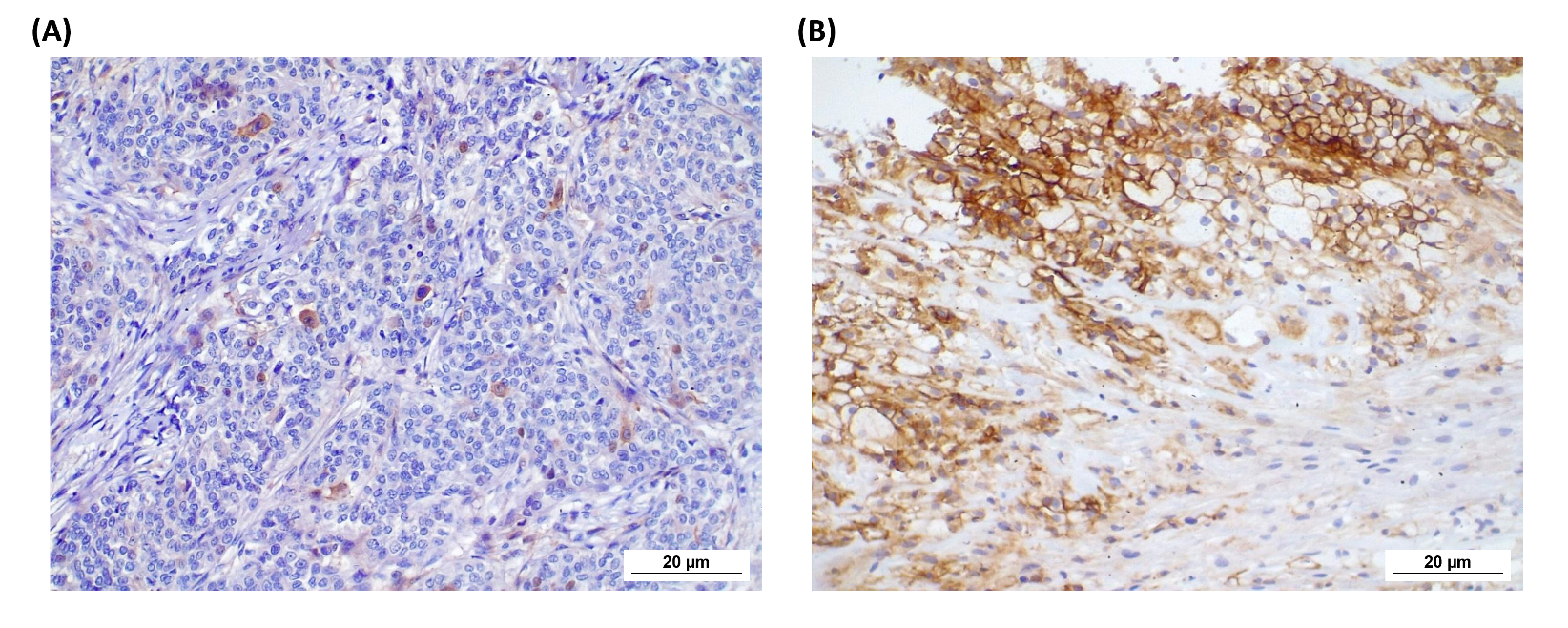
**Supplementary Figure S3. PD-L1 protein expression by immunohistochemistry.** (A) Example of PD-L1 protein expression with an expression lower than 5%, and (B) Example of PD-L1 protein expression with an expression higher than 50%. Abbreviations: PD-L1, programmed death-ligand 1.

**Supplementary Table S4. Cox regression univariate analysis of selected immune and tumor protein markers (*n* = 23)**

| Variables | *n* | **PFS**  **HR (95% CI)** | ***P*** | **OS**  **HR (95%CI)** | ***P*** |
| --- | --- | --- | --- | --- | --- |
| CD8 expression | | | 0.502 | 0.998 | |
| Negative | 2 | Reference |  | Reference |  |
| + | 15 | 1.4 (0.3-6.6) |  | 0.9 (0.2-4.0) |  |
| ++ | 1 | 0.9 (0.1-10.6) |  | 0.9 (0.1-10.0) |  |
| +++ | 1 | 7.0 (0.5-99.3) |  | 0.0 (NA) |  |
| CD8 intensity | | | 0.636 | 0.825 | |
| 0 | 2 | Reference |  | Reference |  |
| 3 | 17 | 1.4 (0.3-6.4) |  | 0.8 (0.2-3.9) |  |
| PD-L1 expression | | | 0.226 | 0.108 | |
| Negative | 4 | Reference |  | Reference |  |
| + | 10 | 0.6 (0.2-2.1) |  | 0.3 (0.1-1.1) |  |
| ++ | 4 | 0.1 (0.0-1.0) |  | 0.1 (0.0-0.6) |  |
| +++ | 1 | 0.3 (0.0-3.0) |  | 0.2 (0.0-2.7) |  |
| PD-L1 intensity | | | 0.453 | 0.167 | |
| 0 | 4 | Reference |  | Reference |  |
| 1 | 1 | 1.0 (0.1-9.0) |  | 0.3 (0.0-3.6) |  |
| 2 | 6 | 0.4 (0.1-1.8) |  | 0.2 (0.0-1.1) |  |
| 3 | 8 | 0.3 (0.1-1.4) |  | 0.2 (0.0-0.9) |  |
| PD-L1 score^a^ | | | 0.045 | 0.099 | |
| 0-3 | 14 | Reference |  | Reference |  |
| >3 | 5 | 0.3 (0.1-1.0) |  | 0.3 (0.1-1.3) |  |

^a^The composite score was calculated by multiplying the level of protein expression by the corresponding staining intensity, providing an integrated measure of marker abundance. Abbreviations: NA, not available; PD-L1, programmed death-ligand 1.

**Supplementary Table S5. Impact of PD-L1 composite score^a^ on response (*n* = 17).**

|  | PR | SD | PD | *P* |
| --- | --- | --- | --- | --- |
| PD-L1 score | | | | 0.011 |
| 0-3 | 0 (0.0%) | 10 (83.3%) | 2 (16.7%) |  |
| >3 | 3 (60.0%) | 2 (40.0%) | 0 (0.0%) |  |

^a^The composite score was calculated by multiplying the level of protein expression by the corresponding staining intensity, providing an integrated measure of marker abundance. Abbreviations: PD, progression disease, PD-L1, programmed death-ligand 1; PR, partial response; SD, stable disease.

**Study protocol**

| 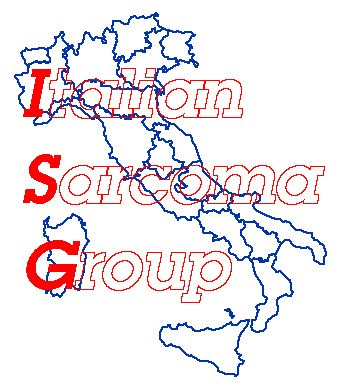 | 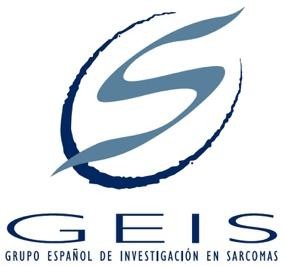 | 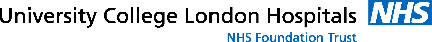 |
| --- | --- | --- |

GEIS-52

**CLINICAL TRIAL PROTOCOL**

Title:

**Phase I-II trial of sunitinib and/or nivolumab plus chemotherapy in advanced soft tissue and bone sarcomas**

Study Acronym: ImmunoSarc 2 Sponsor Protocol Number: GEIS-52 BMS Protocol Number: CA209-754

EudraCT Number: 2016-004040-10

**Protocol Version: 11 of 1 July 2021**

International Sponsor: Grupo Español de Investigación en Sarcomas (GEIS)

**Coordinating Investigators in Spain:**

- Dr. Javier Martín – Hospital Universitario Fundación Jiménez Díaz (Madrid)

**Coordinating Investigator in Italy:**

- Dr. Silvia Stacchiotti – Fondazione IRCCS Istituto Nazionale dei Tumori (Milan)

**Coordinating Investigator in the United Kingdom:**

- Dr. Sandra Strauss – University College London Hospitals NHS Foundation Trust (London)

**Trial summary**

The clear cell sarcoma (CCS) cohort of the ImmunoSarc2 clinical trial consists of a phase II, single-arm, non-randomized, open-label, multicenter prospective international study conducted in Spain, Italy, and the United Kingdom. The study tests the combination of sunitinib plus nivolumab in advanced/metastatic CCS, with an estimated sample size of 23 evaluable patients, and progression-free survival rate (PFSR) at 6 months as the main endpoint.

**Inclusion and exclusion criteria**

*Inclusion criteria*

1. Patients (or legal tutors) must provide written informed consent prior to performance of study-specific procedures and must be willing to comply with treatment and follow-up. Informed consent must be obtained prior to the start of the screening process. Procedures conducted as part of the patient’s routine clinical management (e.g., blood count, imaging tests, etc.) and obtained prior to signature of informed consent may be used for screening or baseline purposes as long as these procedures are conducted as specified in the protocol.
2. Age: 12-80 years.
3. Diagnosis of dedifferentiated chondrosarcoma, extraskeletal myxoid chondrosarcoma, vascular sarcomas (including angiosarcoma, hemangioendothelioma, and intimal sarcomas), solitary fibrous tumor (excluding dedifferentiated SFT), alveolar soft part sarcoma, and clear cell sarcoma confirmed by central pathology review.
4. Mandatory paraffin-embedded tumor blocks must be provided for all subjects without exception for biomarker analysis before treatment.
5. Metastatic/locally advanced unresectable disease in progression in the last 6 months according to RECIST 1.1. Patients with a recent diagnosis of metastatic disease can be eligible (if they are not candidates for anthracycline-based treatment).
6. Patients should have previously received at least anthracyclines. Patients in the cohorts of subtypes sensitive to antiangiogenic therapy (SFT, ASPS, CCS, EMC or DDCS) are eligible even if not previously treated.
7. Previous therapy with antiangiogenics is allowed.
8. Measurable disease according to RECIST 1.1 criteria.
9. Eastern Cooperative Oncology Group (ECOG) Performance Status of 0-1.
10. Adequate hepatic, renal, cardiac, and hematologic function.
11. Laboratory tests as follows:
    - Absolute neutrophil count ≥1,200/mm³
    - Platelet count ≥100,000/mm³
    - Bilirubin ≤1.5 mg/dL
    - PT and INR ≤1.5 in the absence of anticoagulant therapy
    - AST and ALT ≤2.5 times the upper limit of normal
    - Creatinine ≤1.5 mg/dL (or Cr clearance ≥60 ml/min)
    - Calcium ≤12 mg/dL
12. Left ventricular ejection fraction ≥50% by echocardiogram or MUGA scan.
13. Females of childbearing potential must have a negative serum or urine pregnancy test within 7 days prior to enrollment and agree to use birth control measures during study treatment and for 6 months after its completion. Patients must not be pregnant or nursing at study entry. Women/men of reproductive potential must have agreed to use an effective contraceptive method.

*Exclusion criteria*

- - - 1. Four or more previous lines of chemotherapy for the advanced disease.
      2. Previous anti-programmed death-1 (PD-1), anti-programmed death-ligand 1 (PD-L1), anti PD-L2 or anti CTLA-4 antibody.
      3. Prior immune-related adverse event (Grade 3 or higher immune-related pneumonitis, hepatitis, colitis, endocrinopathy) with prior immunotherapy (e.g. cancer vaccine, cytokine, etc.).
      4. Active, known, or suspected autoimmune disease.
      5. A condition requiring systemic treatment with either corticosteroids (> 10 mg daily prednisone equivalents) or other immunosuppressive medications within 14 days of study drug administration. Inhaled or topical steroids and adrenal replacement doses > 10 mg daily prednisone equivalents are permitted in the absence of active autoimmune disease.
      6. Uncontrolled intercurrent illness including (not limited to): symptomatic congestive heart failure (CHF) (New York Heart Association [NYHA] III/IV), unstable angina pectoris or coronary angioplasty, or stenting within 24 weeks prior to registration, unstable cardiac arrhythmia (ongoing cardiac dysrhythmias of NCI CTCAE version 4.0 Grade ≥2), known psychiatric illness that would limit study compliance, intra-cardiac defibrillators, known cardiac metastases, or abnormal cardiac valve morphology (Grade ≥3).
      7. Positive test for hepatitis B virus surface antigen (HBV sAg) or hepatitis C virus ribonucleic acid (HCV antibody), indicating acute or chronic infection.
      8. Other disease or illness within the past 6 months, including any of the following:
         - Myocardial infarction
         - Severe or unstable angina
         - Coronary or peripheral artery bypass graft
         - Symptomatic congestive heart failure
         - Cerebrovascular accident or transient ischemic attack
         - Pulmonary embolism
      9. Evidence of a bleeding diathesis.
      10. Ongoing cardiac dysrhythmias > Grade 2.
      11. Uncontrolled hypertension, defined as blood pressure >150/100 mm Hg despite optimal medical therapy.
      12. Psychiatric illness or social situation that would preclude study compliance.
      13. Pre-existing thyroid abnormality, defined as abnormal thyroid function tests despite medication.
      14. Prolonged QTc interval (i.e., QTc >450 msec for males or QTc >470 msec for females) on baseline ECG.
      15. Hemorrhage ≥Grade 3 in the past 4 weeks.
      16. History of allergy to study drug components.
      17. Previous anticoagulants due to thrombotic events.
      18. History of another cancer with the exception of adequately treated basal cell carcinoma or cervical cancer in situ.
      19. Presence of brain or central nervous system metastases.

**Treatment**

## *Induction phase: Day 1 to Day 14*

For adult patients (≥18 years), an initial induction phase is defined from day 1 to day 14 for all patients, in which only sunitinib will be given as follows:

- Sunitinib 37.5 mg/day orally continuously

For pediatric patients (<18 years) in the induction phase sunitinib will be given at 25 mg/day unless the body surface area (BSA) of the patient is >1.7. If BSA is >1.7, then sunitinib 37.5 mg/day will be given.

## *Maintenance phase: From day 15 onwards*

After the 14-day induction phase, the maintenance phase is defined as follows:

- Adult population: Sunitinib 25 mg/day orally continuously + nivolumab 240 mg every 2 weeks regimen infused over 30 minutes.
- Pediatric population (<18 years):

Weight ≥40 kg: Sunitinib 25 mg/day orally continuously + nivolumab 240 mg every 2 weeks regimen infused over 30 minutes.

Weight <40 kg: Sunitinib 25 mg/day orally continuously + nivolumab 3 mg/kg every 2 weeks regimen infused over 30 minutes.

Treatment will continue until disease progression, development of unacceptable toxicity, non-compliance, withdrawal of consent by the patient, or investigator decision.

**Dose adjustment criteria and guidelines for drug withdrawing or withholding**

*Sunitinib*

During the maintenance phase, sunitinib could be modified in case of toxicity, namely from 25 mg continuously to 25 mg 2 weeks on / 1 week off. However, the sunitinib dose/schedule could be adapted at the investigator’s discretion in case of recurrent or low-grade toxicities (e.g., multiple G2 toxicities), to maintain the best dose intensity.

Sunitinib dose/schedule modifications could be applied to manage treatment-related toxicity that is not controlled by optimal supportive care or not tolerated due to interference with normal daily activities regardless of severity (grade).

Hematologic toxicities:

- Grade 1-2: Continue at the same dose level.
- Grade 3 or intolerable Grade 2: Withhold dose until toxicity is Grade ≤2 or has returned to baseline, then resume treatment at the same dose level.
- If the toxicity recurs with Grade 3 severity (or intolerable Grade 2) at the discretion of the investigator, reduce the dose by 1 level or adapt the schedule.
- Grade 4: Withhold dose until toxicity is Grade ≤2 or has returned to baseline, then reduce dose by one level or adapt schedule and resume treatment.

GCSF or erythropoietin administration is allowed for sporadic use.

Cardiac toxicity (Left Ventricular Ejection Fraction):

- Grade 1-2: Continue at the same dose level.
- Grade 3 or intolerable Grade 2: The dose should be interrupted and/or reduced without clinical evidence of CHF but with an ejection fraction <50% and >20% below baseline.
- Grade 4: The dose should be interrupted and/or reduced without clinical evidence of CHF but with an ejection fraction <50% and >20% below baseline.

Other toxicities:

Exceptions: If CHF, pancreatitis, necrotizing fasciitis, nephrotic syndrome or thrombotic microangiopathy occur, sunitinib should be discontinued.

- Grade 1-2: Continue at the same dose level.
- Grade 3 or intolerable Grade 2: Withhold dose until toxicity is Grade ≤1 or has returned to baseline, then resume treatment at the same dose level.
- If recurrence of Grade 3 or intolerable Grade 2 at the discretion of the Investigator, reduce the dose by 1 level or adapt schedule.
- Grade 4: Withhold therapy until Grade ≤1 or has returned to baseline then reduce the dose by one level or adapt the schedule and resume treatment or discontinuation at the discretion of the investigator.

In order to palliate different side effects (anorexia, diarrhea, vomiting, hypothyroidism, anemia, hypertension, and skin toxicity), symptomatic therapy will be advised.

Re-escalation to the previous dose level could be suggested in the absence of Grade 3 or higher in the case of hematological toxicities or in the absence of Grade 2 or higher in the case of non-hematological toxicities during the previous treatment cycle.

*Nivolumab*

Dose escalations are not permitted for nivolumab.

Dose reductions could be evaluated from a flat dose of 240 mg to 3 mg/kg in those patients with nivolumab-related toxicity and a weight lower than 80 kg.

Nivolumab administration should be delayed for the following:

Any Grade ≥2 non-skin, drug-related AE, with the following exceptions:

- Grade 2 drug-related fatigue or laboratory abnormalities do not require a treatment delay.
- Any Grade 3 skin, drug-related AE.

Any Grade 3 drug-related laboratory abnormality, with the following exceptions for lymphopenia, leukopenia, AST, ALT, total bilirubin, or asymptomatic amylase or lipase:

- Grade 3 lymphopenia or leukopenia does not require dose delay.
- If a subject has a baseline AST, ALT, or total bilirubin that is within normal limits, delay dosing for drug-related Grade ≥2 toxicity.
- If a subject has baseline AST, ALT, or total bilirubin within the Grade 1 toxicity range, delay dosing for drug-related Grade ≥3 toxicity.
- Any Grade ≥3 drug-related amylase or lipase abnormality that is not associated with symptoms or clinical manifestations of pancreatitis does not require dose delay. The Investigator should be consulted for such Grade ≥3 amylase or lipase abnormalities.

Any AE, laboratory abnormality, or intercurrent illness which, in the judgment of the investigator, warrants delaying the dose of study medication.

Recommended treatment modifications for nivolumab:

- Grade 2 pneumonitis: Withhold dose(s) until symptoms resolve, radiographic abnormalities improve, and management with corticosteroids is complete.
- Grade 3 or 4 pneumonitis: Permanently discontinue treatment.
- Grade 2 diarrhea or colitis: Withhold dose(s) until symptoms resolve and management with corticosteroids, if needed, is complete.
- Grade 4 diarrhea or colitis: Permanently discontinue treatment.
- Grade 2 elevation in AST, ALT, or total bilirubin: Withhold dose(s) until laboratory values return to baseline and management with corticosteroids, if needed, is complete.
- Grade 3 or 4 elevation in AST, ALT, or total bilirubin: Permanently discontinue treatment.
- Grade 2 or 3 creatinine elevation: Withhold dose(s) until creatinine returns to baseline and management with corticosteroids is complete.
- Grade 4 creatinine elevation: Permanently discontinue treatment.
- Symptomatic Grade 2 or 3 hypothyroidism, hyperthyroidism, hypophysitis, Grade 2 adrenal insufficiency, Grade 3 diabetes: Withhold dose(s) until symptoms resolve and management with corticosteroids (if needed for symptoms of acute inflammation) is complete. Treatment should be continued in the presence of hormone replacement therapy as long as no symptoms are present.
- Grade 4 hypothyroidism, hyperthyroidism, hypophysitis, Grade 3 or 4 adrenal insufficiency, Grade 4 diabetes: Permanently discontinue treatment.
- Grade 3 rash: Withhold dose(s) until symptoms resolve and management with corticosteroids is complete.
- Grade 4 rash: Permanently discontinue treatment.
- Stevens-Johnson syndrome (SJS) or toxic epidermal necrolysis (TEN): Permanently discontinue treatment.

*Criteria to resume treatment*

Subjects may resume treatment with study drug when the drug-related AE(s) resolve to Grade ≤1 or baseline value with the following exceptions:

- Subjects may resume treatment in the presence of Grade 2 fatigue.
- Subjects who have not experienced a Grade 3 drug-related skin AE may resume treatment in the presence of Grade 2 skin toxicity.
- Subjects with baseline Grade 1 AST/ALT or total bilirubin who require dose delays for reasons other than a 2-grade shift in AST/ALT or total bilirubin may resume treatment in the presence of Grade 2 AST/ALT OR total bilirubin.
- Subjects with combined Grade 2 AST/ALT AND total bilirubin values meeting discontinuation parameters should have treatment permanently discontinued.
- Drug-related pulmonary toxicity, diarrhea, or colitis, must have resolved to baseline before treatment is resumed. Subjects with persistent Grade 1 pneumonitis after completion of a steroid taper over at least 1 month may be eligible for retreatment if investigator allows.
- Drug-related endocrinopathies adequately controlled with only physiologic hormone replacement may resume treatment if investigator allows.

If the criteria to resume treatment are met, the subject should restart treatment at the next scheduled timepoint per protocol. However, if the treatment is delayed past the next scheduled timepoint per protocol, the next scheduled timepoint will be delayed until dosing resumes.

If treatment is delayed or interrupted for >6 weeks, the subject must be permanently discontinued from study therapy.
